# Variation of stress levels, burnout, and resilience throughout the academic year in first-year medical students

**DOI:** 10.1371/journal.pone.0240667

**Published:** 2020-10-15

**Authors:** Richard K. Jordan, Shivam S. Shah, Harsh Desai, Jennifer Tripi, Anthony Mitchell, Randall G. Worth

**Affiliations:** Department of Medical Microbiology & Immunology, University of Toledo College of Medicine & Life Sciences, Toledo, OH, United States of America; Chiba Daigaku, JAPAN

## Abstract

Medical student wellness is of great concern in the health care field. A growing number of studies point to increases in suicide, depression, anxiety, mood disorders, and burnout related to physician lifestyles. Mental health issues commencing in medical school have been suggested to have a significant impact on future physician lifestyle and burnout. Tracking the mental health of medical students at the University of Toledo College of Medicine and Life Sciences (UTCOMLS) with standardized indices will help elucidate triggers of poor mental health. Anonymous surveys were developed and distributed to preclinical medical students at five strategic time points throughout the 2018 2019 academic year. Surveys collected basic demographic information as well as inventories measuring perceived stress, burnout, resilience, and mindfulness. 172 M1s (83 males and 89 females) were included in the study and average response rate for the first 4 (out of 5) surveys averaged 74.8%. M1 males and females had on average increased personal burnout over time with females consistently scoring higher. Both males and females had an increase in stress from August to each subsequent month (p<0.05). Females reported a higher level of perceived stress than males in the beginning and middle of the academic year (p<0.05). Both males and females report a gradual decrease in resiliency throughout the academic year. These surveys demonstrated over half of males and females in medical school reported higher perceived stress scores than their gender-matched peers in the general United States population. Our study strengthens documented trends in resiliency, perceived stress, and burnout amongst medical students. More study in designing targeted approaches to ameliorate these findings in the medical student population is warranted.

## Introduction

Medical student mental health is a topic of increasing concern as burnout rates and depression have been on the rise [1]. Moreover, poor mental wellness outcomes among physicians in the workplace are becoming more prevalent. With increasing research in this area, it’s become clear that mental health issues arise in medical school and may have a significant impact on future mental wellness of physicians [2].

A recent systematic review of 195 articles illustrated an overall prevalence of 11.1% for suicidal ideation in medical students, and among students who screened positive for depression, only 15.7% sought treatment [3]. In another large study with a 35% participation rate from all medical students nationwide, 44.6% of medical students scored high on the burnout index, 28.0% reported an intermediate burnout level, 58.2% screened positive for depression, and 9.4% of medical students had suicidal ideation within the past 12 months. Finally, 57.7% of medical students reported high fatigue [4]. Poor mental health potentially has adverse consequences on the ability to learn, subsequently impacting academic performance [5]. These data present an alarming picture, especially given the high rates of subsequent depression, burnout, and suicide amongst American physicians [6].

There are multiple proposed reasons why indicators of poor mental health are more prevalent amongst the general medical student population. The rigor of current medical education, curricular format, the belief that students must be ‘strong’ enough to handle stress to succeed, and the lack of attention given to mental health in comparison to physical health problems are all contributing factors [4].

The current literature is controversial regarding sex discrepancies in mental health among medical students. In a large national longitudinal study of a cohort of medical students surveyed in 2010, Hadman et al. analyzed the risk of depressive symptoms and mental health burden among women at the start of medical school, as compared to their male counterparts [7,8]. They reported that female medical students experienced a higher mental health burden compared to males. These findings were alarming given this was a group of first-year medical students, and other studies suggest that their mental health will continue to decline [5]. Additional studies comparing depressive symptoms by sex are inconclusive, showing either no difference by sex or higher rates among females [9].

Finding positive relationships and effective interventions is important to improve the wellness of medical students. This will allow them to reach their full potential academically, clinically, and personally. The goal of our study is to better understand the trends and variations of stress, burnout, and resilience throughout the academic year in first-year medical students at the University of Toledo College of Medicine and Life Sciences (UTCOMLS). Further, we aimed to understand the relationship between sex and mental health burden in medical school. Data collected from this study serves as a driving force to provide evidence-based interventions supporting medical students in preventing or overcoming mental health challenges and mitigating the risk of depression and burnout. The survey instrument will continue to be administered throughout medical students’ training at the UTCOMLS to allow a longitudinal understanding of time points in the curriculum where the mental health of students may be at highest risk.

## Methods

A cross-sectional study of 700 students at the UTCOMLS were asked to complete a 53-question survey. The survey was optional, and responses were kept anonymous. There was no incentive attached to completing the survey. The only criteria that had to be met to participate in this study was enrollment in the UTCOMLS MD program. This study was conducted after approval from the UTCOMLS Institutional Review Board (IRB). Our data focused on the first-year medical students’ (M1) responses to the survey.

**Fig 1:**
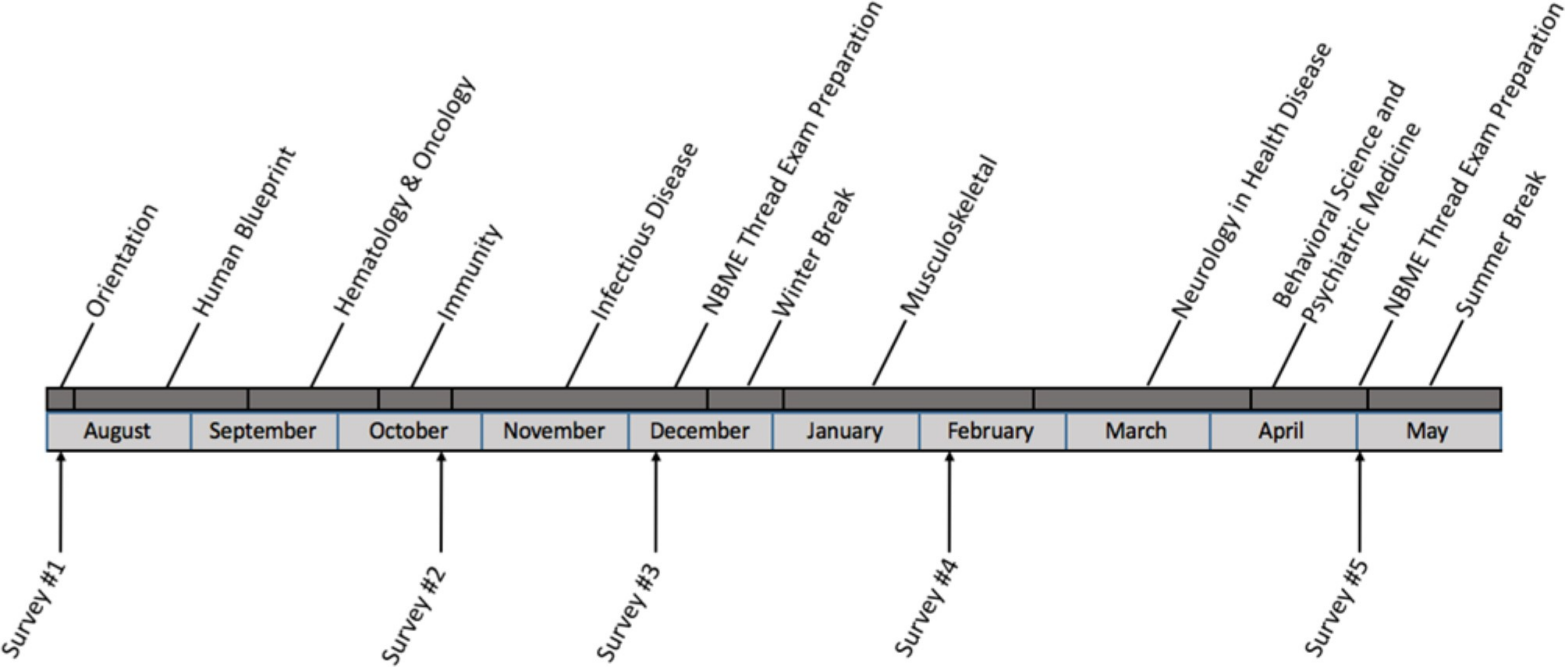
Timeline of survey completion and medical school curriculum.

Data collection was completed using an electronic survey emailed to all students at five set time points during the year. The survey was sent to M1s in August, the beginning of the academic year. They received subsequent surveys in October, December, February and May. These dates correlated with various events during the preclinical medical school curriculum (Fig 1: Timeline of Survey Completion and Medical School Curriculum).

Validated scales were chosen to provide objective insight into the students’ resiliency, burnout, and stress. The survey tools consisted of the Brief Resiliency Scale (BRS), Copenhagen Burnout Inventory (CBI), and Perceived Stress Scale (PSS) [10–12]. The BRS contains 6 questions regarding resilience. The scores are stratified as 1.00–2.99 being low resilience, 3.00–4.30 being normal resilience, and 4.31–5.00 being high resilience. A random population of 844 healthy and diseased adults had an average score of 3.7 (normal resilience), which we used as our average general population resilience score for comparison to our study group [13].

The CBI contains 3 parts covering different aspects of burnout including personal burnout, work burnout, and client burnout. Client burnout was excluded from our survey as it did not relate to our population. A recent study exhibited average values of 41.9 and 54.5 for personal burnout in males and females, respectively [14]. Work burnout showed averages of 47.4 and 48.7 for males and females, respectively [14]. These average values were used to compare our study group to the general population.

The PSS has a total of 10 questions which are graded on a 5-point scale with some questions having a reversed grading order. The PSS showed an average of 12.1 and 13.7 for males and females, respectively from a sample of 2,387 respondents [15]. These average values served as the comparison value to our study group.

Statistical analysis for this project was completed using SPSS Statistics 24. The primary tests used were descriptive statistics and an independent samples t-test with Levene’s Test for Equal Variance. The data was stratified by sex. A one-way ANOVA test was performed to understand the longitudinal trends across the academic year in males and females. If significant effects were observed, Least Significant Difference (LSD) post-hoc comparisons were performed as appropriate.

## Results

**Fig 2:**
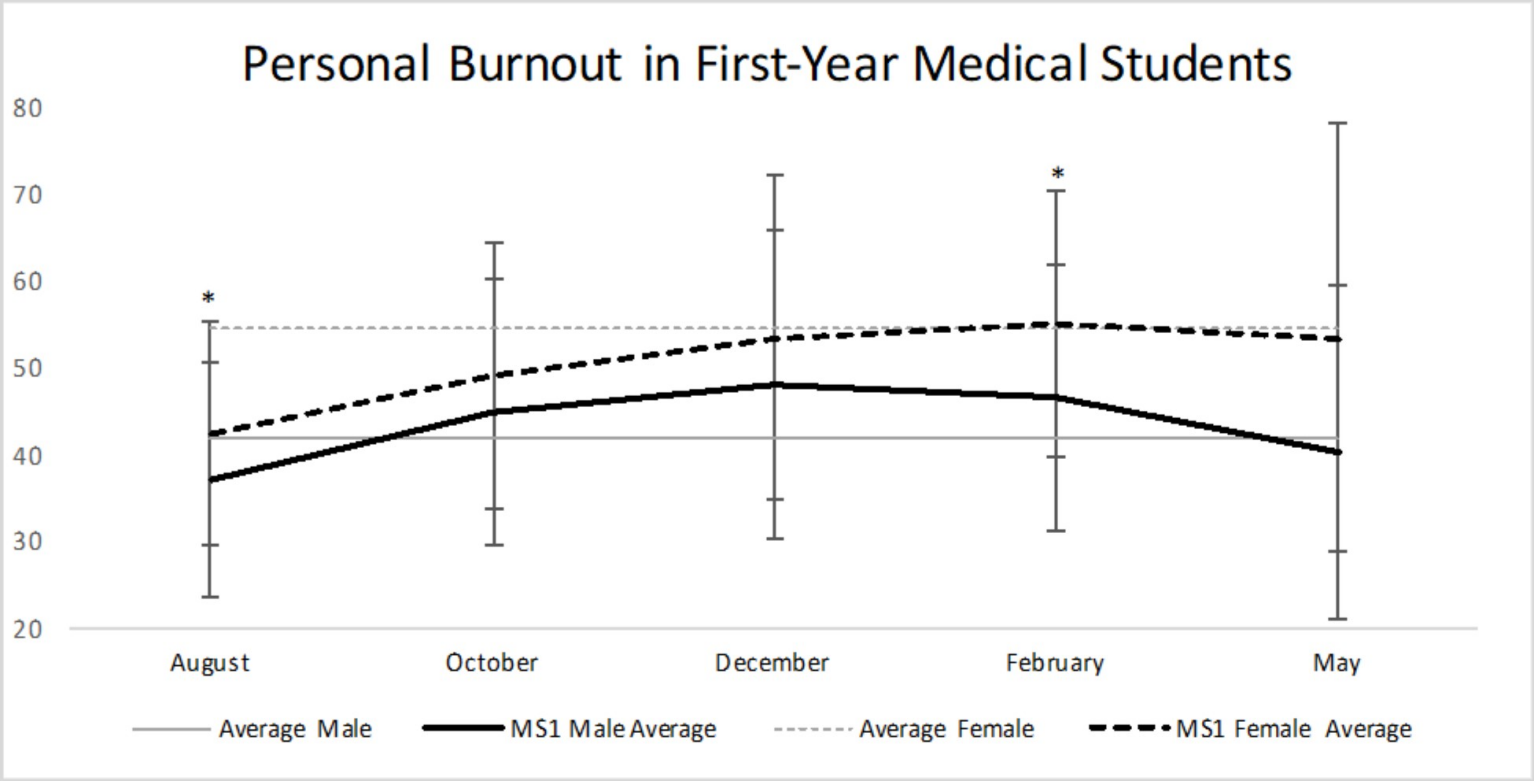
Personal burnout in first-year medical students. M1 males and females exhibited increased personal burnout over time, but leveled off towards the end of the year. Females were consistently more burned out than males at all five time points. There was a statistically significant difference in burnout during August and February, with females being more burned out (orientation in August, musculoskeletal practical in February). Average male (41.9) and average female (54.5) in general population [14] * = p<0.05.

172 M1s (83 males and 89 females) were included in the study (average age = 23.98, Table 1: General Demographics). Females showed significantly elevated levels of personal burnout (mean of 42.23; SD = 12.90, N = 88) compared to males (mean of 37.03; SD = 13.58, N = 80) upon the initial survey administered in August (orientation) of their first year of medical school (Table 2, Fig 2). Females also had a statistically significant higher personal burnout in February (musculoskeletal practical) (mean of 54.92; SD = 15.42; N = 89) compared to males (mean of 46.5; SD = 15.42; N = 75). Male and female students displayed increasing rates of personal burnout through the first-year curriculum. The LSD post-hoc test revealed that in males there was a statistically significant (p<0.05) increase in personal burnout from August to October, December, and February, but not for May. Similarly, in females there was a statistically significant increase (p<0.05) in personal burnout when comparing August to all subsequent months [14].

**Table 1:** General demographics.

Year in Medical School	M1
Average Age (years)	23.98
Number of Males	83 (48.3%)
Number of Females	89 (51.7%)

**Table 2:** Personal burnout, work burnout, perceived stress, and resiliency in first-year medical students.

	M1 Male			M1 Female			
	Mean	SD	N	Mean	SD	N	Significance
**Personal Burnout**							
August	37.0313	13.58663	80	42.2348	12.90348	88	0.012*
October	44.6314	15.37779	52	48.9754	15.45333	61	0.138
December	47.9762	17.94632	35	53.4091	18.85517	44	0.198
February	46.5	15.42443	75	54.9242	15.41706	89	0.001*
May	40.2778	19.32684	12	53.3854	24.68406	16	0.14
**Work Burnout**							
August	49.152	6.43359	80	48.6203	7.21245	88	0.616
October	46.0165	16.64145	52	48.0094	15.92559	61	0.517
December	50.9184	8.09527	35	48.2143	6.42118	44	0.102
February	49	16.94138	75	53.8961	15.50272	89	0.056
May	38.9881	19.64778	12	52.0089	22.84854	16	0.126
**Perceived Stress**							
August	11.65	5.77884	80	13.9659	6.19872	88	0.013*
October	19.4808	3.74946	52	20.2623	3.33117	61	0.243
December	16.2571	6.20883	35	17.0682	6.57125	44	0.578
February	15.7333	6.18047	75	18.1023	6.72111	89	0.021*
May	20.4375	3.18264	12	19.25	2.52713	12	0.126
**Resiliency**							
August	3.8133	0.68392	80	3.6723	0.80929	88	0.123
October	3.8109	0.72912	52	3.6612	0.66316	61	0.256
December	3.7143	0.76330	35	3.6743	0.59167	44	0.759
February	3.7622	0.62311	75	3.5417	0.77478	89	0.041*
May	3.7361	1.13587	12	3.4271	1.00732	16	0.454

*Statistical significance = p<0.05.

**Fig 3:**
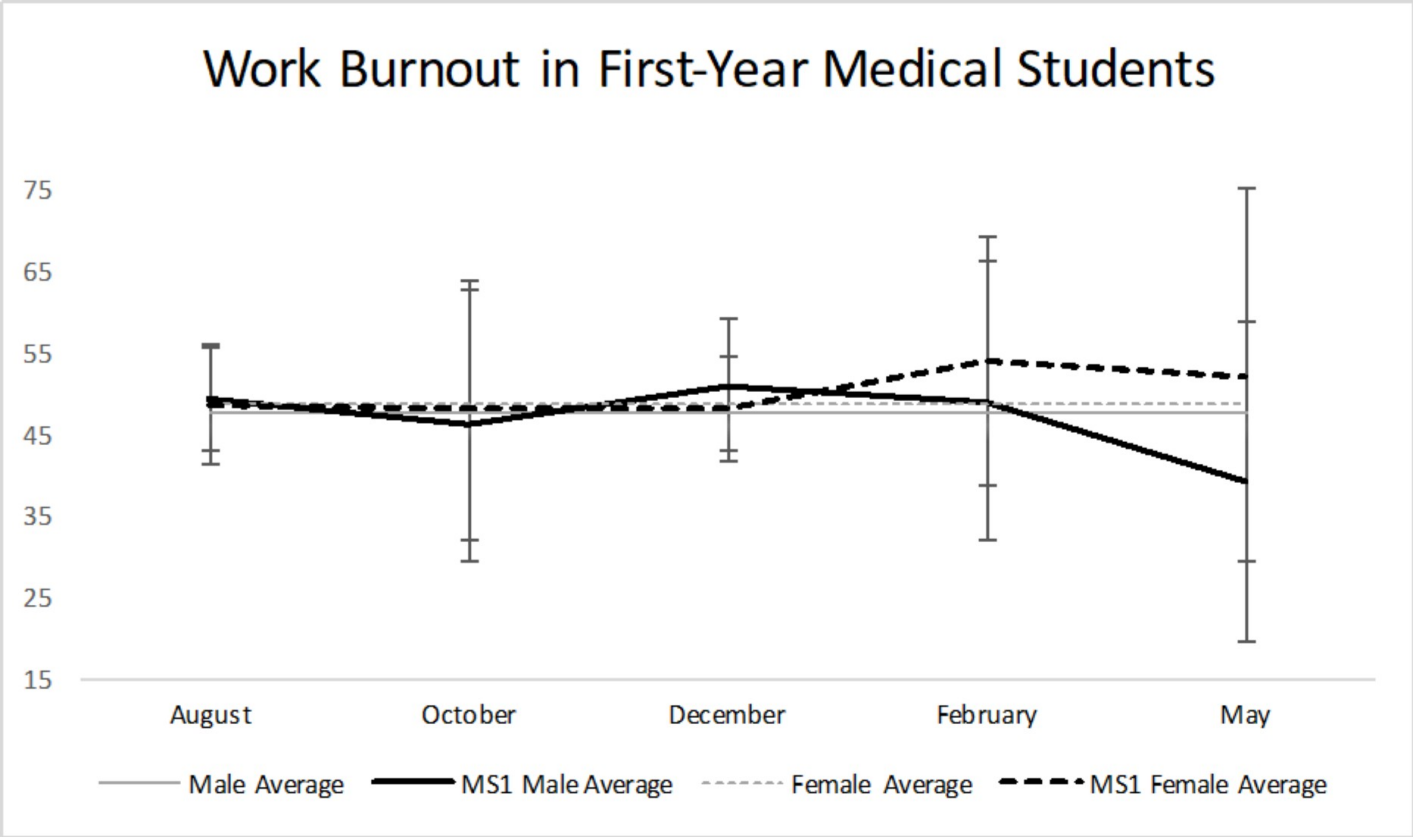
Work burnout in first-year medical students. There were no statistically significant differences in work burnout across all five time points between M1 males and females. Males peaked work burnout in December, while females peaked in February. Average male (47.4) and average female (48.7) in general population [14].

For work burnout (Table 2, Fig 3), there were no statistically significant findings across all five time points between M1 males and females. Males had peaked work burnout in December (NBME thread preparation) at 50.92, while females peaked in February at 53.90. The LSD post-hoc test illustrates that in males there was no statistically significant change in work burnout from August to February, but a statistically significant decrease (p<0.05) in burnout from August to May. That may be explained by the decrease in the May response rate. For females, there was a statistically significant increase (p<0.05) in burnout from August to February [14].

**Fig 4:**
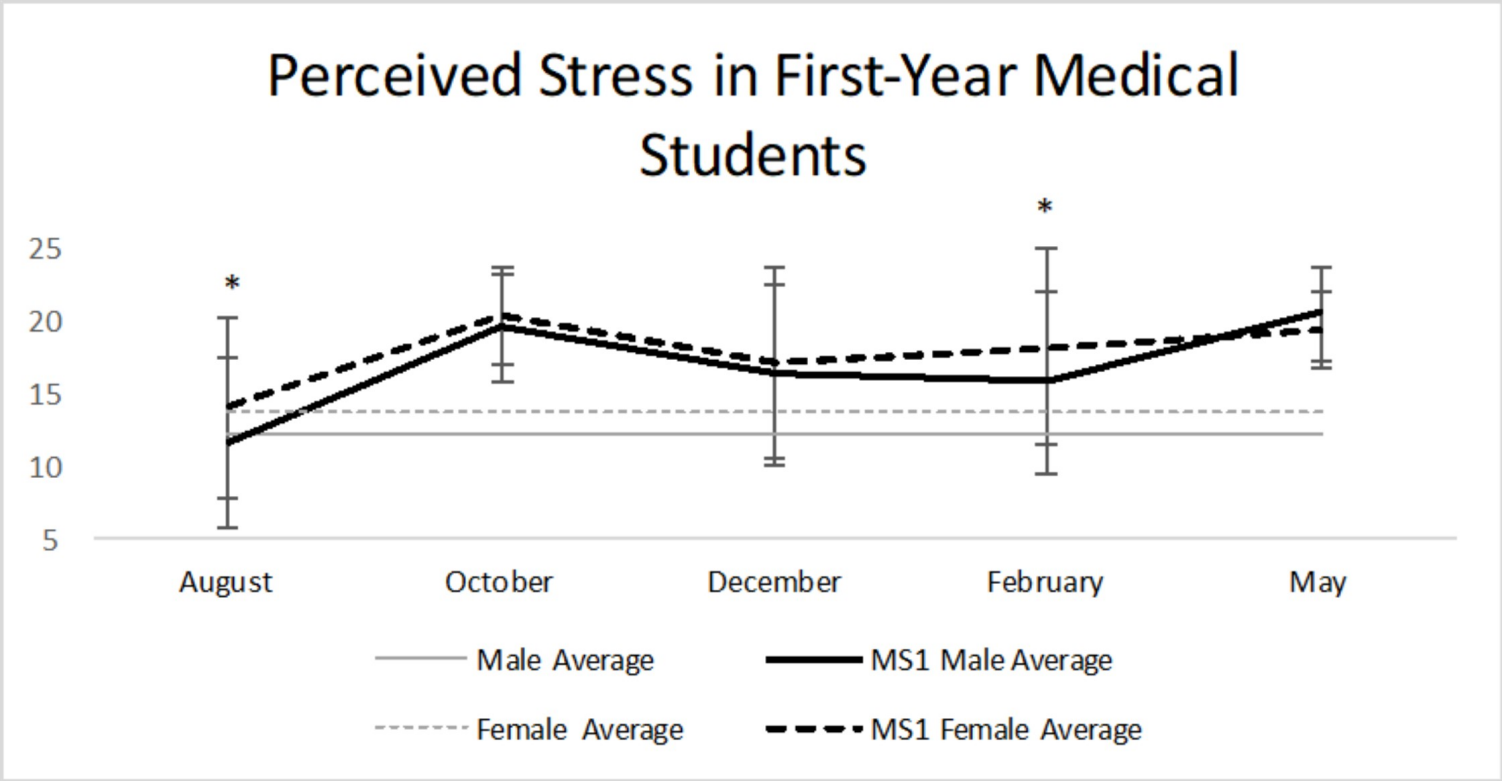
Perceived stress in first-year medical students. M1 females had a statistically significant greater perceived stress during the months of August (orientation) and February (musculoskeletal practical). Average male (12.1) and average female (13.7) in general population [15]. * = p<0.05.

For perceived stress (Table 2, Fig 4), M1 females have a statistically significant greater perceived stress during the months of August and February. M1 males had a mean of 11.65 (SD = 5.78, N = 80) and females had a mean of 13.97 (SD = 6.20, N = 88) in August. This was a statistically significant difference (p = 0.013). For both males and females, perceived stress increased in February. Males had a mean of 15.73 (SD = 6.18, N = 75) while females had a mean of 18.10 (SD = 6.72, N = 88), which showed a statistically significant difference (p = 0.021). The LSD post-hoc test revealed that in both males and females there was a statistically significant increase (p<0.05) in stress from August to each subsequent month [15].

In August, 25/88 (28.41%) females and 23/80 (28.75%) males had perceived stress scores greater or equal to half of a standard deviation than the gender-matched peers in the U.S. general population. This was the only month when less than half of the respondents were not at least a half standard deviation greater than the gender-matched peers. In October, 57/61 (93.44%) of females and 46/52 (88.46%) of males and in December, 24/44 (54.55%) of females and 14/35 (40.00%) of males had a perceived stress score greater or equal to a half standard deviation than the gender-matched peers. In February, 60/88 (68.18%) of female and 37/75 (49.33%) of male students had a perceived stress score greater or equal to a half standard deviation than the gender-matched peers. In May, 15/16 (93.75%) of female and 11/12 (91.67%) of male students had a perceived stress score greater or equal to a half standard deviation than the gender-matched peers.

**Fig 5:**
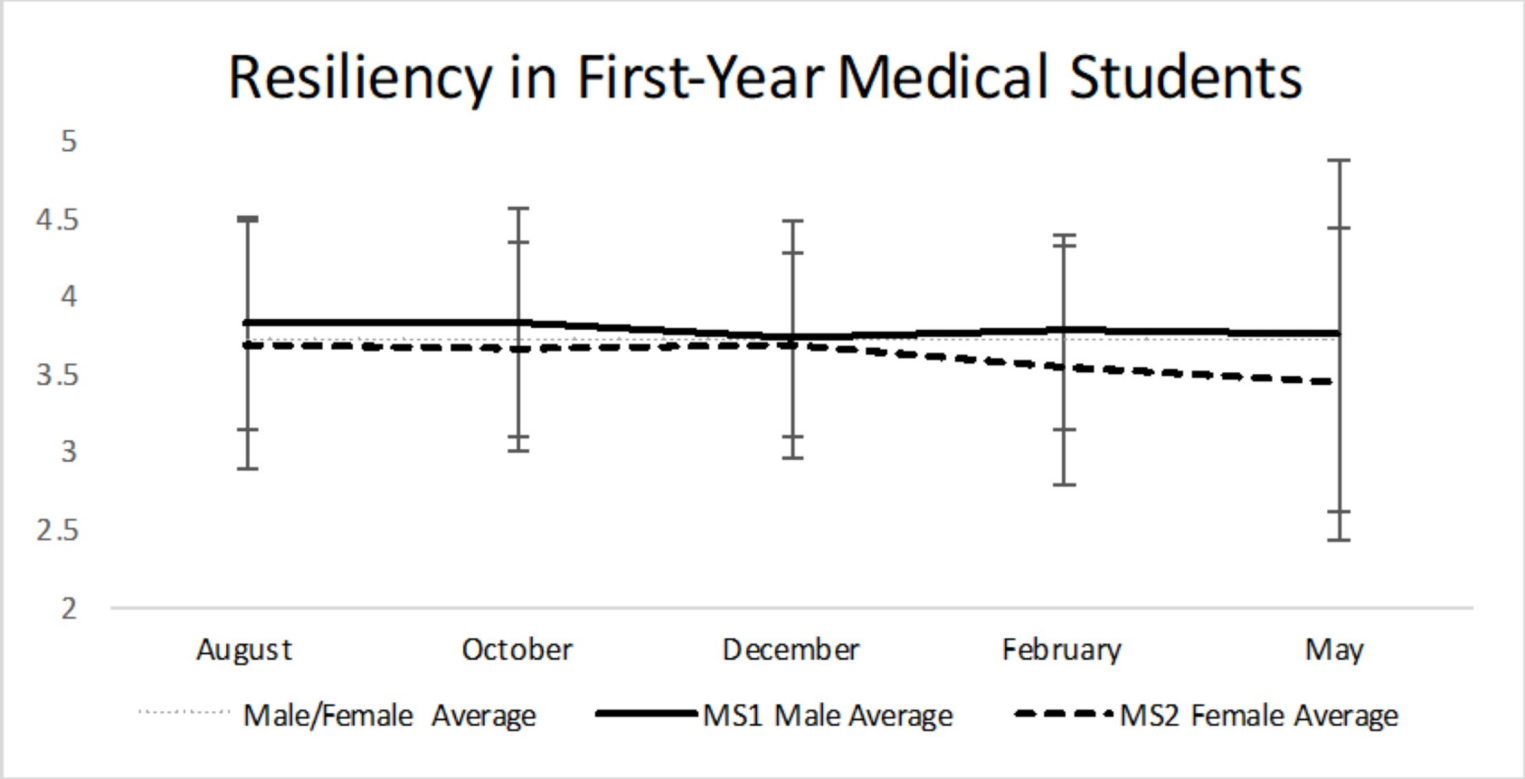
Resiliency in first-year medical students. Both males and females remain in the normal resilience group throughout all five time points, however none reach high resilience (Ranges: 1.00–2.99 = Low resilience, 3.00–4.30 = Normal resilience, 4.31–5.00 = High resilience). M1 females exhibited lower resiliency than males at all time points (statistically significant at the February time point). Notice that males and females had a gradual decrease in resiliency throughout the academic year. Average score of 3.7 from a random population of 844 adults [13]. * = p<0.05.

For resiliency (Table 2, Fig 5), both males and females scored between 3.00–4.30 over all five time points (considered normal resilience by the authors of the BRS). However, females exhibited lower resiliency than males at all five time points, and this is statistically significant in February (p = 0.041). Males had a mean of 3.7622 (SD = 0.62311, N = 75) versus females who had a mean of 3.5417 (SD = 0.77478, N = 88) in February. Throughout the academic year, both males and females have gradually decreased resiliency. It is important to note that the response rate gets progressively smaller throughout the year, and therefore the power to detect a difference diminishes. The LSD post-hoc test illustrates that there is no statistically significant change in resilience from August to each subsequent month in both males and females [13].

## Discussion

It is well known that many medical students experience stress and decreased wellness throughout medical school [16]. Dyrbye and his colleagues studied medical students at seven medical schools in the United States, which showed 49.6% of students experienced burnout and 11.2% reported suicidal ideation within the past year. Importantly, burnout, quality of life, and depressive symptoms at baseline predicted suicidal ideation over the following year [17]. This suggests the need to prevent burnout and depression, and promote a balanced life in an effort to maintain a positive mental health state. Despite these findings, few studies have sought to understand how, when, and why this occurs.

Our study’s goal was to identify variation and patterns of stress levels, burnout, and resilience in first-year medical students over the course of the academic year. Further, we attempted to understand if there was a discrepancy in outcomes by sex. The surveys were administered five times over the course of the year, in hopes to gain an understanding of when medical students appear more at risk for burnout and psychiatric illness. Importantly, we associated the M1 curriculum with the dates that our survey was completed by the students (Fig 1). By knowing when students are more vulnerable to medical school stresses, more resources can be targeted to prevent negative outcomes during those times.

Many studies have demonstrated high prevalence of burnout among medical students. Mazurkiewicz and colleagues studied 86 entering third-year medical students at Mount Sinai School of Medicine in New York which showed 71% met criteria for burnout. This suggests that medical students are often burned out before reaching their clinical clerkships [18]. Additionally, a study of the trends in burnout among 1,098 medical students from three Minnesota medical schools showed 45% experienced burnout, with an increasing trend among clinical students versus preclinical students. Our study used the Copenhagen Burnout Inventory to measure personal and work burnout levels [11]. M1 males and females both exhibited an increasing trend in personal burnout over time, which then stabilized towards the end of the academic year. Females were consistently more burned out than males at all five time points (Table 2, Fig 2). There was a statistically significant difference in burnout during August (p = 0.012) and February (p = 0.001), with females being more burned out. Perhaps females had a more difficult time with adjusting to medical school and its demands. During February, the students were preparing for their musculoskeletal practical, which perhaps led to greater personal burnout in females as it requires many hours spent studying not only textbooks, but also in the cadaver lab. For work burnout, both males and females had similar burnout scores throughout the year, with no statistically significant difference at all five time points (Table 2, Fig 3).

Previous studies have shown that medical students have higher levels of perceived stress and less resiliency than the general population. Perceived stress levels reported by these studies indicated that ≥ 50% of medical students scored at least half of a standard deviation greater than the norm for age-matched peers in the U.S. general population [19,20]. Our study provides further support to these findings. The average M1 male and female perceived stress levels were at least half of a standard deviation greater than the norm for gender-matched peers in the U.S. general population in four of the five months (October, December, February, and May) [10]. This means that in October, December, February, and May over half of the respondents reported stress levels substantially higher than that of age matched peers in the U.S. population. In August and February, the M1 females had a statistically significant elevation in perceived stress when compared to the M1 males (Table 2, Fig 4).

The finding that female medical students have higher levels of perceived stress has been reported internationally, but this has not been consistent. Two Pakistani studies, a Swedish study, a Canadian study, and a U.S. study reported higher levels of perceived stress in females compared to males [21–24]. To the contrary, a Finnish study and a British study did not find a gender difference in perceived stress [25,26]. Our study provides further support that females experience more stress than males, at least during their first year of medical school. These findings are important, as we know that student stress causes depersonalization and reduces empathy [27,28]. Medicine is governed by the Hippocratic oath of doing no harm to patients, and we postulate that this oath should also be applied to training physicians. Ideally, medical students should graduate with the same sense of altruism, compassion, and empathy that they had upon starting.

Resiliency is defined as the ability to remain positive despite experiencing adversity. Students who have greater resiliency possess a mindset and skill set that allows them to work through and overcome adversity [21]. A previous study of Canadian medical students demonstrated lower resiliency than age and gender matched peers in the general population [22]. Commonly, male students have higher resiliency scores than females, however this finding is not unanimous [21,29–31]. Our study provides further support to male medical students possessing higher levels of resiliency than females, at least during the first year of medical school. In all five surveys, the M1 males had higher levels of resiliency than the M1 females, with a statistically significant difference seen in February. However, both M1 males and females experienced a gradual decrease in resiliency throughout the academic year (Table 2, Fig 5). Importantly, according to the authors of the BRS, a score of 3.00–4.30 is considered normal resilience, and both males and females remained in this group at all five time points. However, they never reach high resilience, which is a score of 4.31–5.00.

Fortunately, resiliency is modifiable, and has recently been designated as a priority for medical education [31]. Building resiliency may be key to the reduction of stress and protection against the effects of stressors that arise during medical school. While our study group exhibited normal resilience throughout the academic year, it may be beneficial for them to gain skills, so they have high resilience, therefore protecting them from medical school stresses. Our findings highlight that resilience of medical students continues to be an important area for future study, and medical schools should focus on developing students’ resiliency.

Despite the overwhelming evidence of high stress and burnout in medical students, research is lacking in finding strategies to promote resiliency, health, and wellness to prevent such negative experiences. Unfortunately, an associated stigma has prevented medical students from seeking mental health services when needed, which further complicates our ability to be proactive in preventing these health issues [32].

A few studies have revealed strategies to be beneficial to medical students. A cross-sectional study of medical students at the University of North Dakota School of Medicine and Health Sciences found that greater use of approach-oriented coping strategies rather than avoidant-oriented strategies was associated with significantly decreased risk of burnout (p = .02) and was inversely correlated with depression [32]. This suggests that adequate coping strategies promotes mental health resilience. Furthermore, another study found that self-reported engagement in self-care activities had an inverse relationship with perceived stress and direct relationship with quality of life in medical students, suggesting that students who maintain a balanced life sustain greater resiliency [19].

Mentorship programs led by faculty of the medical school may be a good approach to reducing burnout and alleviating stress in students. Jordan et al. studied the utility of a resident-student mentorship program for fourth-year medical students during their emergency medicine subinternship [33]. The Maslach Burnout Inventory was completed by the intervention and control group before and after the rotation. The intervention group had a statistically significant higher personal accomplishment score after the rotation. Most students also felt the program positively impacted their rotation, decreased stress, provided career guidance, and positively impacted their personal and professional development. A mentorship program is a relatively easy resource to incorporate into medical training and appears to have many positive implications.

The practice of mindfulness may also be a promising intervention to help ameliorate anxiety and stress, and enhance academic performance in medical students. Mindfulness-Based Stress Reduction (MBSR) is a behavioral intervention designed to teach self-regulatory skills for stress reduction and emotional management. There is an overall lack of data to support the use of MBSR in medical students, and studies that have been conducted contain a small sample size making it difficult to make any conclusions. However, mindfulness meditation has been supported in many other healthcare professionals and students. A study of nursing professionals and students who underwent mindfulness and loving kindness meditation over a six-week period revealed a significant reduction (p<0.05) between pre-intervention and post-intervention scores for perceived stress, burnout, depression, and anxiety [34]. However, there was no significant difference between post-intervention and follow-up which suggests the need to consistently practice mindfulness to maintain the benefits. Qualitative results showed “improvement in the reactivity to inner experience, a more attentive perception of internal and external experiences, and greater attention and awareness of actions and attitudes at every moment”. A meta-analysis of medical, nursing, social work, psychology, and other health students found that MBSR decreases stress, anxiety, depression, and improves mindfulness, mood, self-efficacy, and empathy [35]. Finally, a study completed in Ireland examined first- and second- year medical students’ perception and satisfaction ratings of a seven-week MBSR program [36]. The M1 (n = 140) class had mandatory participation, whereas it was optional for the M2 (n = 88) class. M1 students were less satisfied with the content and learning outcomes than M2 students (p < .0005). The M1 class provided feedback that they hoped for less discussion and more practice. The second-year medical students’ satisfaction with the program suggests that an optional program for all medical students may be beneficial for medical schools to integrate into their training programs.

Drolet et al implemented a program at Vanderbilt that takes a comprehensive view of wellness [37]. They implemented focusing on multiple dimensions of wellness (emotional, physical, and mental) and engages students through leadership, class building, and a longitudinal curriculum with active workshops. St. Louis University, among others including U. Toledo, have implemented curricular changes in hopes of alleviating burnout and providing a more robust educational experience. Some facets included in St. Louis University’s updated curriculum include Pass/Fail grading, reduced contact hours for the first two years, longitudinal electives, and implementing learning communities [38]. Importantly, improved results were reported after just two years of implementation thus providing strong evidence for longitudinal studies of medical education during curricular changes.

There were many limitations to our study. First, this was a questionnaire-based study, so reporting bias cannot be ignored and certainly may contribute to variation. Additionally, the surveys were completed by medical students at a single medical school in the United States, so it is more difficult to generalize the results to all medical students. However, our students are selected from a national pool of applicants and the curriculum is similar in nature to many other programs so we believe our data will be generalizable. Moreover, measures of burnout, interpersonal reactivity and tolerance for ambiguity used by AAMC graduate questionnaire show that our school is the same as “All Medical Schools” further supporting that our data may be generalizable. Future studies may incorporate multiple medical schools from across the world, which would increase the sample size and therefore power of our study. Importantly, the surveys were completed anonymously, so we do not know which students completed the survey at each time point. It is possible that a different set of students responded to each survey, and therefore our longitudinal data is invalidated. Future studies would ideally assign each student a number that they record into each survey so we can be certain we are following the same students throughout the academic year. Additionally, our response rate decreased as the year progressed, and therefore selection bias may be present. Finally, although this study measured levels of burnout, stress, and resiliency, specific information about the particular burnout triggers and stressors experienced was not collected. There are also strengths of our study including frequency of surveys and response rate. Our survey is sent to M1, M2, and M4 students five times during the academic year and to M3 students at the end of each clinical clerkship. This allows us to detect points in the curriculum that have a negative impact on student wellness and informs curricular changes or offer additional support to students during these times. Moreover, we have an excellent response rate allowing us to interpret the data as a good representative of our class and not just a small cohort. Although response rate decreased near the end of the year, we’re working on ways to improve response rates thus empowering the study.

It is clear that medical schools must be more proactive in preventing student burnout and psychological disorders, while promoting resiliency. Our findings support the paradox that medical education actually fosters unhealthy habits, psychological distress, and burnout in first-year medical students. Additionally, our data suggests that females experience more burnout, perceived stress, and lower resilience than males. Though some stressors may be inevitable, such as academic pressure, this does not preclude the utility of stress reduction programs. Additionally, our data provides insight into time points during the academic year that students perceive as more stressful. If medical schools gather similar data, this can guide them in altering the curriculum accordingly, or increasing stress reduction resources during those times. The stigma that prevents many students from seeking mental health care when necessary has created a barrier that we must overcome. Promoting resiliency strategies through a mentorship program, mindfulness practice sessions, or classes on the importance of maintaining a balanced life and utilizing healthy coping strategies appears to be the next best step forward but will require an institutional cultural change in order to empower students’ participation.

## Supporting information

S1 File Data medical student 6-3-2019. (CSV)
